# Robot-assisted early mobilization of intensive care patients: a feasibility study protocol

**DOI:** 10.1186/s40814-022-01191-0

**Published:** 2022-11-05

**Authors:** Angelika Warmbein, Ines Schroeder, Amrei Mehler-Klamt, Ivanka Rathgeber, Jana Huber, Christina Scharf, Lucas Hübner, Marcus Gutmann, Johanna Biebl, Andreas Lorenz, Eduard Kraft, Michael Zoller, Inge Eberl, Uli Fischer

**Affiliations:** 1 Clinical Nursing Research and Quality Management Unit, University Hospital LMU Munich, Marchioninistr. 15, 81377 Munich, Germany; 2 Department of Anaesthesiology, University Hospital LMU Munich, Marchioninistr. 15, 81377 Munich, Germany; 3 Professorship of Nursing Science, Faculty of Social Work, Catholic University of Eichstätt-Ingolstadt, Kapuzinergasse 2, 85072 Eichstätt, Germany; 4 Department of Orthopaedics and Trauma Surgery, Musculoskeletal University Center Munich (MUM), University Hospital LMU Munich, Marchioninistr. 15, 81377 Munich, Germany

**Keywords:** Robotics, Unit, Intensive care, Early mobilization, Feasibility study, Care, Nursing

## Abstract

**Background:**

Early mobilization positively influences the outcome of critically ill patients, yet in clinical practice, the implementation is sometimes challenging. In this study, an adaptive robotic assistance system will be used for early mobilization in intensive care units. The study aims to evaluate the experience of the mobilizing professionals and the general feasibility of implementing robotic assistance for mobilization in intensive care as well as the effects on patient outcomes as a secondary outcome.

**Methods:**

The study is single-centric, prospective, and interventional and follows a longitudinal study design. To evaluate the feasibility of robotic-assisted early mobilization, the number of patients included, the number of performed VEM (very early mobilization) sessions, and the number and type of adverse events will be collected. The behavior and experience of mobilizing professionals will be evaluated using standardized observations (*n* > 90) and episodic interviews (*n* > 36) before implementation, shortly after, and in routine. Patient outcomes such as duration of mechanical ventilation, loss of muscle mass, and physical activity will be measured and compared with a historical patient population. Approximately 30 patients will be included.

**Discussion:**

The study will provide information about patient outcomes, feasibility, and the experience of mobilizing professionals. It will show whether robotic systems can increase the early mobilization frequency of critically ill patients. Within ICU structures, early mobilization as therapy could become more of a focus. Effects on the mobilizing professionals such as increased motivation, physical relief, or stress will be evaluated. In addition, this study will focus on whether current structures allow following the recommendation of mobilizing patients twice a day for at least 20 min.

**Trial registration:**

ClinicalTrials.gov, NCT05071248. Date: 2021/10/21

## Background

Many studies have shown positive impacts of very early mobilization (VEM) on the functional and cognitive health [1–7] of intensive care unit (ICU) patients. It achieves the best possible rehabilitation [8, 9] and shortens the length of stay in the ICU and hospital [3].

It has also been described that VEM can prevent functional disorders [9, 10]. Regular mobilization, meaning all forms and processes of mobilization aiming at the rehabilitation of intensive care patients, leads to important positive healing processes and consequently to an overall faster recovery [11]. Assisted walking movements in particular reduce the risk of decubitus ulcers, maintain mobility and cardiac function, and facilitate bowel movements. These mobilizing measures are already part of the therapy programs of less seriously ill patients [9, 12].

However, optimal VEM therapy, i.e., mobilization starting within 72 h of ICU admission, should include daily mobilization sessions of at least 20 min, combining verticalization and gait-like leg movements. Due to the critical physical conditions of intensive care patients, VEM therapy can therefore only be carried out with an extraordinarily high level of personnel effort, especially if the patient is ventilated [13]. Often, critically ill patients cannot stand on their own feet due to their severe limitations and have to be “exercised” on a therapy device. The transfer of intensive care patients from bed to a separate therapy device is time-consuming and risky for patients. Therefore, this method is not often performed in clinical practice. The current S2e guideline (“Positioning therapy and early mobilization for prophylaxis or therapy of pulmonary dysfunctions” [14]) recommends active mobilization to be performed by at least two qualified staff members. For these and many other reasons, such as sedation/paralysis of the patients concerned (46%), unconsciousness (4%), staff shortage (17%), weekend (8%), etc. [15], only a quarter of the eligible patients are currently early mobilized [8, 16]. This has considerable consequences/significant impacts on the healing process, the burden on relatives, and the costs incurred by health insurances [10] and insured people.

Currently, several devices on the market allow automated robotic early mobilization therapy. Compared to manual early mobilization, robotic support has the advantage that mobilization in bed can reduce the risk of falls for patients. In addition, the physical strain for mobilizing professionals is reduced as the robotic device takes over verticalization and leg movement. Some models verticalize and mobilize patients simultaneously. However, this requires a patient’s transfer from their bed to the training device and then back to the bed.

The MobiStaR project (Mobilization of intensive care patients by a new standard in adaptive robotics) is based on the development model of complex interventions of the Medical Research Council (MRC) [17]. In a cycle of piloting, evaluation, implementation, and (further) development, the framework for the use of the early mobilization device is created within the overall duration of the project.

The early mobilization robot used in our study design is able to verticalize the patient in their bed without transfer. Additionally, it generates a movement of the legs while measuring and supporting the patient’s own movement. The device fulfills the requirements for mobilizing critically ill patients in an intensive care unit, maintains hygiene standards, and provides the best possible support for the patient’s own movement. However, the path towards a nursing robot that can be used in a standardized manner for all eligible, critically ill patients strongly depends on the environment, the processes, and the organizational procedures in which the robot is integrated. If it fulfills the requirements of sustaining the quality of care and significantly improves patient outcomes and their chances of recovery, thereby relieving the personnel, and is economically attractive, it simplifies the integration into an ICU. There is currently no adequate evidence for the benefit of the use of robotics in the early mobilization of ICU patients.

Evidence-based data is currently lacking on whether the use of robotic-assisted early mobilization can improve patient outcomes, what the experience of users is like, and whether the organizational and structural implementation in the daily routine of an intensive care unit is possible.

## Methods/design

### Aim

The aim of this interventional study is to determine if robotic-assisted early mobilization of critically ill patients is feasible and useable. In addition, it is intended to identify the effects of this form of VEM compared to conventional, manual VEM on the experience of the mobilizing profession and the outcomes of the patients.

To achieve this purpose of the study, the following research questions will be examined in the context of (1) organizational feasibility, (2) evaluation of effects on patient outcomes, and (3) evaluation of the mobilizing professionals’ experience.

### Study design

The present study is a mixed-methods, single-centric, prospective intervention study with a comparison to actual standard therapy and takes place in anesthesiological intensive care units of a university hospital in southern Germany (Table 1).

**Table 1:** Study design

	Aim	Design	Participants	Estimated sample size
**Effects on patient outcomes**	Comparison of patient outcomes with robot-assisted very early mobilization (VEM) to conventional VEM	Interventional with comparison to a historic patient group	Intensive care patients	Approx. 30 patients per group
**Effects on behavior and experience of the mobilizing professionals**	Comparison of the emotions and the behavior with robot-assisted VEM (2 evaluations) to conventional VEM	Qualitative interviews and standardized observations	Mobilizing professionals (nurses, physiotherapists, physicians)	Observations *n* = 90–150, interviews *n* = 36 depending on data saturation
**Organizational feasibility**	Evaluation of the feasibility and integration in the ICU	Standardized observations	Nurses, physiotherapists	Approx. *n*=210–300

### Participants

#### Patients

The study population consists of patients undergoing elective surgical procedures and scheduled for postoperative treatment in the anesthesiological intensive care unit. Patients will be included in the prospective intervention study according to the following inclusion criteria: the surgical intervention and postoperative care and therapeutic treatment in the ICU are planned, and the preoperative patient consents in writing for the study. The expected duration of ventilation is more than 48 h. The patients are older than 18 years, their weight is between 45 and 135 kg, and their height is between 1.50 and 1.95 m. Exclusion criteria are chronic bedriddenness, a clinical frailty scale ≥ 7 [18], chronic ventilation (more than 24 h) before admission to the intensive care unit, pregnancy, elevated intracranial pressure/risk for elevated intracranial pressure/recent cerebral hemorrhage, pre-existing neuromuscular disease resulting in chronic limitation of strength and performance, and a sternotomy during a surgical procedure.

Patients within the historical comparison group will be retrospectively selected within the same criteria. If they met any of the exclusion criteria during their intensive care unit stay, they will not be included in the historical group. No matching of the interventional and historical groups is planned.

#### Mobilizing professionals

The mobilizing professionals consist of physicians, nurses, and physiotherapists working in anesthesiological intensive care units and are regularly involved in mobilization. An employment contract at the Hospital is required for all professional groups/mobilizing professionals. Physicians, nurses, and physiotherapists will be included according to inclusion and exclusion criteria.

Nurses with advanced training in anesthesia and intensive care and/or nurses who have at least 3 years of professional experience in an intensive care unit will be included. In addition, these persons have an employment contract at Hospital. Similarly, specialists in leading positions in intensive care units with completed residency training meet the inclusion criteria. Additionally, physiotherapists with at least 3 years of professional experience in an intensive care unit will be included. For T_2_ and T_3_, all specialists should also be assigned to the anesthesiological intensive care units. Specialists will only be included if they have given consent to participate in the study.

Persons that are members of the MobiStaR project team, have less than 3 years of professional experience as a nurse or specialist in an ICU, or are still in residency training will be excluded. Physiotherapists with less than 3 years of professional experience in intensive care units are also excluded. Individuals who are not employees of the Hospital are also excluded. In T_2_ and T_3_, specialists who are not assigned to the anesthesiological intensive care units according to the duty schedule are excluded.

### Patient sample size

In order to test correlations using multiple-variate models (multiple linear regressions) with a statistical power of 80% on approximately 8 independent variables (IV) compared to the dependent variable (DV), an approximate total sample size of 50 patients is required. Thus, with an expected drop-out of 10%, 55 patients (robotic intervention and historical comparison group) should be included in the study. A sample size of 20 subjects is considered a lower limit with moderately strong associations between IVs and DV and inclusion of a maximum of 5 IVs, with alpha=5% and power=80% [19, 20]. In this regard, if 30–35 patients are included in the robot-assisted intervention and a maximum of 6 IVs, meaningful results can be expected to be obtained in a manageable period of time. The study is completed as soon as the required number of patients has been recruited for the intervention. In 2020, we provided a recruitment estimation to ensure achieving the calculated sample size. The corresponding number of cases for the historical group will be taken from the routine data.

Interviews and observations will be performed at three different points in time to assess the behavior and experience of mobilizing professionals. Interviews will be carried out with at least four persons of every professional group (physicians, nurses, physiotherapists) until data saturation occurs. The approximate sample size for interviews is *n*=36 for all time points of evaluation. At any point in time, between 30 and 50 mobilizations will be observed, so a total number of *n*= 90–150 observations are planned. The mobilizing professionals can be observed multiple times. All participants included in the study may withdraw their consent to participate in the study at any time.

## Procedures and data collection

### Study plan

The study covers the period of early mobilization by the robotic system of patients who meet the inclusion and exclusion criteria. These are mobilized with the robotic early mobilization device approximately twice a day for 20 min, or at least 10 times within 7 days. The data collection will take place for 5 to 6 months, beginning in September 2021. Three study series will be performed during the study period: (1) feasibility line of robot-assisted VEM in the ICU, (2) care line: behavior and experience of the mobilizing professionals (evaluating conventional early mobilization before intervention), and (3) prom line: effects on patient outcomes.

### Evaluation plan

All patients will receive a physical examination at different time points to assess physical functionality and muscle strength, as well as a sonographic examination of leg muscles, diaphragm, and lungs. These examinations and the collection of clinical scores will be performed on day −1 (preoperatively); on postoperative days 1, 2, and 3, then once a week if the patient remains in the ICU; on day 28; on the day of discharge from the ICU; and on a follow-up examination within the context of routine examinations approximately 3 months after discharge from the ICU [21]. The follow-up examination should only take place if the patients present themselves at the LMU Hospital due to medically indicated follow-up examinations (not study-related). Alternatively, patients can be asked about their condition by telephone. Patient-related interventions and conducting the informed consent interviews are carried out by the study physicians.

The evaluations of the behavior and experience of the mobilizing professionals and the feasibility line will be collected accompanying the robotic-assisted mobilizations of the patients. The survey ends with the last robotic-assisted mobilization. Observations of the professionals will only be performed with patients who have given consent to participate in the study. The informed consent of mobilizing professionals and evaluation will be conducted by nursing scientists and study physiotherapists.

The feasibility of the study will be rated when the intervention group has at a minimum equivalent effects on the patient outcome as the historical comparison group, a minimum rate of robot-assisted mobilizations (i.e., 50%) can be performed, no serious adverse events occur, and feasibility is judged to be acceptable by the users.

### Description of variables and tools employed in the evaluation of the variables

A unique three-digit ID will be assigned to each patient, under which all data will be recorded pseudonymously. All invasive procedures performed on patients will be carried out as routine procedures independently of the study in the ICU according to medical indication. The following study-related procedures will be performed on the patients beyond the informed consent and documentation of patient-related data. All required information collected in routine clinical practice is to be obtained from the patient documentation system (electronic patient record) (Table 2).

**Table 2:** Parameters used for the evaluation of the effects on patients’ outcome

**Clinical examination**		
Strength level of upper and lower extremities at last physical examination using Medical Research Council classification (scale: 0–5)		
**Sonographic examination**		
Diaphragm	M. quadriceps femoris	
**Personal data**		
Age (years)	Sex (m/f/d)	Height (cm)
Weight (kg)	Diagnosis	Chronic conditions
**Laboratory values from clinical routine in last 24h**		
**Medication**		
Sufentanil (μg/d)	Insulin bolus + infusion (IU)	
Piritramide (mg/d)	Naloxone (mg/d)	
Midazolam (mg)	Sodium picosulfate (mg/d)	
Dexmedetomidin (μg/d)	Neostigmine (mg/d)	
Propofol (mg/d)	Macrogol 3350 (units/d)	
**Clinical data**		
SOFA score	Operative status	
APACHE II score	Min. Horovitz index (mmHg)	
SAPS II score	max. PEEP (mbar)	
RASS score	max. Pinsp (mbar)	
VAS score	max. respiratory rate (x/min)	
GCS score	Data organ replacement (yes/no)	
Temperature (°C)	ECMO therapy (yes/no)	
Mean arterial pressure (mmHg)	Duration of invasive ventilation since admission (hours)	
Heart rate (bpm)	Duration of non-invasive ventilation since admission (hours)	
Cardiac index (L/min)	Duration of invasive ventilation since admission (0.0–1000.0)	
Bicarbonate (mmol/L)	Export urine (mL)	
Blood pH	AKIN stadium	
Lactate (mmol/L)		
Amount of reflux (mL)		
Bowel movement (active/sluggish /none)		
Amount of stool (frequent/average/little)		

Within the episodic interviews [22], the stress, motivation, and physical strain of the mobilizing professionals will be evaluated. The focus is on the experienced emotions within the mobilization situations. The distress thermometer [23] will be used in conjunction with each interview. The behavior and attitude of mobilizing professionals will be observed using standardized observation schemes [24]. In addition, the following data will be included in robotic-assisted mobilization: ventilation (yes/no), medication (in the weaning process ➔ yes/no; analgosedation ➔ yes/no; catecholamine ➔ yes/no), gender, weight, and height of the patient. Observation will only occur during the mobilization of patients who have given consent to participate in the study.

For rating feasibility, minimum and maximum rates are used and shown in braces behind the parameters. Data on recruitment (number of newly admitted and eligible patients, the enrolled (min. 50%) and excluded patients), as well as retention rates (the number of patients with discontinued interventions/adverse events (max. 10%)/severe adverse events) and type of adverse events (caused by professionals, patients, or the device) are documented each week. In addition, the following intervention-related data will be collected: duration and set-up time of the intervention (max. 25 min in mean), number of mobilizing persons (<2 persons), degree of verticalization, minutes in the highest degree of verticalization, steps per minute, and minutes of intervention in total (20 min in mean), as well as maximum hip angle. The mobilizing professionals will rate physical stress (max. 3 in mean) and feasibility (min. 4 in mean) on every early mobilization that is performed on a 7-point Likert scale.

### Intervention

#### Robot-assisted early mobilization

**Fig. 1:**
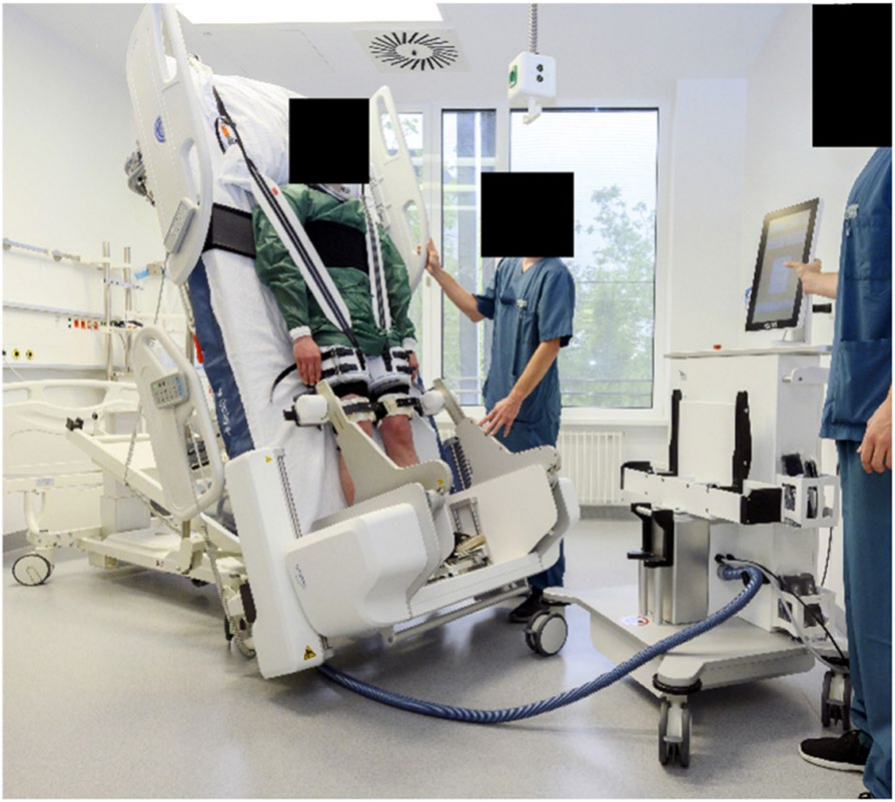
Training of a robot-assisted mobilization (University Hospital LMU Munich 2020)

Patients included in the study, according to the inclusion and exclusion criteria, will be mobilized using the robotic system by the nursing ward team. The aim is to perform a standardized mobilization with verticalization within the first 72 h after admission to the intensive care unit (Fig. 1). If possible, this should be performed twice a day for 20 min, with a minimum of 10 treatment cycles over 7 days. Treatment characteristics such as timing, intensity, duration, and complications will be documented.

Robot-assisted early mobilization is performed only if it is deemed safe according to the criteria and recommendations of the Consensus Conference [25]. This Consensus Manuscript provides recommendations on the conditions under which safe active mobilization is feasible in ventilated patients. It considers four categories (respiratory, cardiovascular, neurological, others). In this study, patients should only be robot-assisted if this is in accordance with the recommendations of the traffic light system [26] level green or yellow. Level green indicates a low risk of an adverse event, and the yellow level shows potential risks and consequences of an adverse event, but the potential benefits of mobilization outweigh the risk. The criteria are discussed with the ward team prior to each robotic-assisted mobilization. Since transferring to a therapy device as described is not required for mobilization with the VEMO© system, the mobilization is categorized as “in-bed-exercise” (versus out-of-bed mobilization). The patients can be verticalized within the bed up to 70°. Here, a leg movement can be generated according to gait patterns.

The mobilizing professionals are trained for 90 min in robotic-assisted mobilization with healthy respondents. From every participating ward, 9 nurses are participating in the training. Product specialists accompany the professionals in mobilizing study patients until they feel safe to operate the device by themselves. The research team accompanies the mobilizing professionals through the whole study in every mobilization. Users can be certified as super users who are qualified to train other nurses or physiotherapists within the use of the device, so that there is a snowball system in knowledge.

#### Conventional early mobilization

The comparison group is a historical collective, which also meets the inclusion and exclusion criteria of the study. These patients were early mobilized following the ward routine of the intensive care unit’s conventional early mobilization according to the instructions of the treatment team, consisting of physicians, nurses, and physiotherapists. Conventional early mobilization cannot be precisely defined based on a retrospective study [27]. The information used for the study regarding early mobilization and the defined outcome criteria of the patients is taken from the routinely collected data.

##### Education and informed consent

Only patients who are capable of giving consent and can be informed preoperatively will be included by the study physicians. Informed written consent will be obtained from all patients who meet the inclusion criteria. If the patients withdraw their consent at a later point in time, they will be asked whether the data collected up to this point in time may still be used. Otherwise, all data collected up to that point will be destroyed. There is no intention to include persons from the group of persons in need of special protection.

##### Clinical examination to determine physical function/health-related quality of life

To assess physical function and muscle strength, the following non-invasive examinations will be performed, and/or scores will be collected as required by the study:

FSS-ICU [28]: the FSS-ICU assesses the patient’s “Physical Performance” based on the following 5 factors: turning, transition from lying to sitting, transition from sitting to standing, sitting at the edge of the bed, and walking. For each of the 5 tasks, a minimum of 0 to a maximum of 7 points can be assigned.

At the follow-up examination approximately 3 months after discharge from the ICU, the health-related quality of life will also be assessed using the SF-36 questionnaire [29].

##### Sonographic examination of the lungs, diaphragm, and musculus quadriceps femoris

By means of ultrasound, the following parameters are evaluated in the course of the study:

The diaphragm is characterized by determining the diaphragm thickness, the thickening fraction, and the motility. The musculus quadriceps femoris is characterized by determining its thickness and by using the cross-sectional area. The methodology of the ultrasound examination is described in detail in the literature [30–34].

The treatment team does not differ for the individual patients; it usually consists of nurses from the corresponding intensive care units, assigned physiotherapists, and the corresponding ward physicians. For the duration of the study, an additional study team will be established, consisting of study physicians, study nurses, and technical support from the manufacturer.

Robotic-assisted early mobilization should be performed within the first 72 h postoperatively, if possible, and should be performed at least twice a day for 20 min until the seventh postoperative day or at least 10 cycles of treatment during the intensive care unit stay. The frequency of treatment, treatment duration, and intensity are recorded. Treatment-associated events will be recorded. In case of hemodynamic, respiratory, or other instability during treatment, the therapy session can be discontinued at any time. The decision to discontinue mobilization rests solely with the treatment team. The study team can advise and act in accordance with the Declaration of Helsinki.

Within biweekly meetings of the operative working research team, the results and the ongoing of the study are evaluated.

### Data analysis

In the context of organizational feasibility, descriptive data is reported and visualized for robotic-assisted VEM. When minimum/maximum rates are reached, further analysis will be performed. Otherwise, the intervention is not seen as feasible. Subsequently, regression analyses are used to contextualize and quantify the data. Depending on the variables and the type of distribution, they are quantified after calculating degrees of freedom, and correlations are tested using applicable analysis methods.

Interview data on stress experience and physical behavior will be collected within a robot-assisted mobilization situation and evaluated and visualized by means of qualitative content analysis [35]. Data of the distress thermometer will be analyzed by means of descriptive statistics [23]. The observations of positioning and mobilization behavior of the mobilizing professionals will be analyzed using descriptive statistics [36].

Within the study population, conventional early mobilization of critically ill intensive care patients (historical comparison group) will be compared with robot-assisted early mobilization. The data will be evaluated by graphical representations of the individual parameters in the course by means of box plots and scatter diagrams. Associations between parameters are quantified using appropriate (depending on scale level and distribution) correlation coefficients. For comparison between conventional and robot-assisted VEM, commonly used robust statistical methods are applied.

After 50% of the participants have been included in the study, the first interim analysis will be performed and is the basis for further decisions.

### Data management (data protection, anonymization, data storage)

For the entire project, an overarching data protection statement Art. 6 DSVGO (General Data Protection Regulation) of the data protection officer of the LMU Hospital is available (Procedure Number 1582a of 13 July 2021).

The data will be collected by means of digital questionnaires. The patients will receive a three-digit pseudonymized ID after giving their consent. Target criteria collected in routine clinical practice will be recorded with the routine case number and transferred to the research database created specifically for the project. Data monitoring is done inhouse by a biostatistical and bioinformatical institute. After completion of the documentation, the case number will be replaced by the above-mentioned ID. All personal data will be recorded under this ID. The data from the survey forms are promptly stored electronically in a secure folder. These are secured by the network of the participating institutions, and access to the data is restricted.

Only the research team has access to the research database. Access to personal data (effects on patient outcomes) is restricted to the study physicians, who are bound by medical confidentiality. After the individual patient has completed the study, the personal reference is removed, and the encryption code is only kept in a written document in a lockable cabinet in the anesthesiological ICU, to which only the clinical study director has access. Decoding is only performed for the safety of the patients (= medical reasons) or in case of a change of the scientific question (= scientific reasons). The regulations of medical confidentiality and data protection are observed in this study. Patients will be informed in detail about data protection during patient education. Access to study-related data is only possible via the respective study directors. All data will be destroyed according to the usual retention periods (Federal Data Protection Act).

Only the study team of the LMU Hospital and the Catholic University of Eichstätt-Ingolstadt (experience and behavior of the users) has access to the collected data.

The names of the participants and all other confidential information are subject to confidentiality and the regulations of the DSVGO and the Federal/State Data Protection Act (BDSG/BayDSG). Data of the study participants will not be passed on. Third parties will not be given access to the original documents. The data collected during the study will be kept until the data analysis is completed and then destroyed. Pseudonymized data may be shared with scientific project partners as part of the discourse on the study.

### Ethical considerations

The study is designed as a clinical intervention study with comparison to a historical patient population. Patient participation is voluntary. The value of early mobilization in critically ill patients has been proven, as has the safety of early mobilization. Harmful events occurred very rarely in comparable studies, and serious adverse events seen in association with the study did not occur [5, 13]. The use of the VEMO© system has also been studied and found to be safe. Thus, participating patients have no a priori disadvantage. The VEMO© system has a CE certificate and is approved for the early mobilization of critically ill patients. It is categorized as a class 2a medical device and is in regular use in several German and international hospitals. The system is only used for the approved indication (early mobilization of critically ill patients). A hygiene concept for the application of the system was developed in cooperation with the hospital hygiene department. The surveys within the scope of organizational feasibility accompany the interventions and pose no risk to patients through the observational function.

Otherwise, the study team has no influence on the treatment of the patients.

The primary benefit in terms of effects on patient outcomes is to determine if robot-assisted mobilization differs from conventional early mobilization in its ability to reduce ventilation time, muscle atrophy, and ICU-acquired weakness. Individual patients could benefit from intensive robot-assisted early mobilization in terms of shorter ventilation, less muscle atrophy, and better physical functionality. A lasting negative impact on the patient group is not expected if treatment is performed with a safe, non-invasive medical device and intensive physiotherapeutic exercise. Serious adverse events associated with the medical device are not known. Possible adverse events such as short-lasting changes in blood pressure and heart rate, the accidental removal of drains, or the development of skin lesions due to the mobilization cuffs could occur.

From the data obtained, improved therapy concepts can be developed, and the use of robot-assisted mobilization can be established as part of a new standard of care. This study makes a significant contribution to the future improvement of therapy for critically ill patients. If the measures of robot-assisted VEM prove superior to those of historical, conventional VEM, the new treatment technique could be quickly implemented into the clinical routine in ICUs based on this study.

The data collected during the study is not available to the treating physicians during the patient recruitment phase. This way, no negative influence on the therapy of the individual patient can arise. Even if conclusions regarding the treatment of future patients or patients from other ICUs cannot be drawn from the data obtained in an unlimited and uncritical manner, the study presented here provides a valuable gain in knowledge with the aim of comparatively examining different forms of early mobilization of the effect on a specific patient population. By simultaneously surveying the experience and behavior of the mobilizing professionals, it is also possible to record their workload when using the new therapy. Given the high workload in ICUs, a feasibility study is essential, which is why the study focuses on users, patients, and structures. All participants in this study (patients and professionals) gave informed and written consent to the interventions before including in the study.

In summary, this study makes an important and necessary contribution to improving the therapy of critically ill patients. There are no study-related burdens for the individual patients, and participation in the study is without risks for the patients.

### Obligation of the study management according to the study protocol

The study directors, as well as all participating scientists, commit themselves to conduct the study described herein in accordance with the study protocol. Changes to the study protocol are only possible after consultation with the ethics committee; if necessary, a new evaluation will be obtained. In case of severe adverse events or violation of the participants, the study will immediately be stopped by the study management.

## Discussion

This evaluation will provide new insights for implementing a robotic device into ICUs, concerning patient outcomes and the feasibility as well as potential effects for mobilizing professionals. If the effects on patients show a better rehabilitation outcome concerning muscle loss and routine parameters, this new technology might reduce the duration in intensive care and in the hospital. Since the device can also be used to mobilize sedated or immobile patients at an early stage, there is a chance that mobilization frequency can be increased. Small studies have already shown the benefit of early mobilization in the care of strokes [12, 37]. The implementation relies on the adaption of organizational structures in a highly organized setting and may lead to additional work for the mobilizing professionals. Regarding the evaluation of behavior and experience of mobilizing professionals, it will survey whether early mobilization becomes a stronger focus of work due to the technical device and whether personal effects such as increased motivation or stress occur. Moreover, the use of the robotic system might reduce lifting (work) for nurses and physiotherapists, which could imply physical relief. In addition, the study will also focus on the feasibility of implementation and whether the current structures allow following the recommendations of mobilizing patients twice a day for at least 20 min.

The aim of the study is to evaluate whether a new standard of care can be implemented in the intensive care setting with a robotic system and whether the setting with its current structures could implement this standard of care permanently. For this, it is essential that at least the same outcomes of mobilization are generated for patients, that the mobilizing professionals experience a positive effect, and that time and staff retention can be implemented in the intensive care unit.

### Limitations

The study is performed with a small number of patients. Results in patients who experience other serious illnesses may differ. The study is single-centric, so the data depend on staff and patients, and external validity is reduced. The survey of effects on patient outcomes will mainly examine parameters recommended in the literature. Possible other parameters might show other effects. The results of the interviews of the mobilizing professionals might vary due to the qualitative approach. The observations are performed by several researchers, which might influence the continuity of data.

## Dissemination plan

The results of the study, as well as results from the respective surveys, will be made available to the public subsequently. This will be enacted in the form of publications and in contributions on scientific conferences.

## Data Availability

The datasets generated or analyzed during the current study are not publicly available due to incompletion but will be available from the study directors on reasonable request.

## Abbreviations

VEM: Very early mobilization
ICU: Intensive care unit
MobiStaR project: Mobilization of intensive care patients establishing a new standard in adaptive robotics project
MRC: Medical Research Council
LMU Hospital: Ludwig-Maximilians-University Hospital Munich
IV: Independent variable
DV: Dependent variable
ID: Identificator
SOFA Score: Sepsis-related Organ Failure Assessment score
APACHE II Score: Acute Physiology And Chronic Health Evaluation II score
SAPS II Score: Simplified Acute Physiology Score II
RASS Score: Richmond Agitation-Sedation Scale
VAS Score: Visual Analog Scale
GCS Score: Glasgow Coma Score
Max. PEEP: Maximum positive end-expiratory pressure
Max Pinsp: Peak inspiratory pressure
ECMO therapy: Extracorporeal membrane oxygenation therapy
AKIN Stadium: Acute Kidney Injury Network Stadium
VEMO system: Very Early Mobilization Operator System (name of the robotic system used in the study)
FSS-ICU: Functional Status Score for intensive care units
SF-36 questionnaire: Short Form questionnaire for health-related life quality with 36 questions
DSVGO: Datenschutzgrundverordnung, German General Data Protection Regulation
CE Certificate: “Communauté Européene” Certificate; European certificate for medical devices
